# Implications of Indolethylamine N-Methyltransferase (INMT) in Health and Disease: Biological Functions, Disease Associations, Inhibitors, and Analytical Approaches

**DOI:** 10.3390/brainsci15090935

**Published:** 2025-08-28

**Authors:** Seif Abouheif, Ahmed Awad, Christopher R. McCurdy

**Affiliations:** 1 Department of Medicinal Chemistry, College of Pharmacy, The University of Florida, Gainesville, FL 32610, USA; s.abouheif@ufl.edu (S.A.); a.awad@ufl.edu (A.A.); 2 UF Translational Drug Development Core, The University of Florida, Gainesville, FL 32610, USA

**Keywords:** indolethylamine N-methyltransferase, DMT, neurotransmitter metabolism, sigma-1 receptor, INMT inhibitors

## Abstract

Indolethylamine N-methyltransferase (INMT) is a Class 1 methyltransferase responsible for N-methylation of various endogenous and exogenous compounds, including tryptamine, serotonin, and dopamine. This review aims to provide a comprehensive overview of the biological and therapeutic relevance of INMT, emphasizing the human isoform (hINMT), highlighting its structural characteristics, disease association, and recent advances in analytical strategies. Dysregulation of INMT activity has been linked to a range of pathological conditions, including neuropsychiatric disorders, neurodegeneration, and several forms of cancer. These associations are addressed by integrating current findings across disease pathophysiology, enzyme inhibition, and analytical methodologies, including both radiolabeled and non-radiolabeled in vitro assays, for measuring INMT activity. We further explored the chemical diversity of INMT inhibitors, both natural and synthetic, and highlighted key compounds with therapeutic relevance. Additionally, recent commercial assays for quantifying INMT activity are emphasized. By integrating emerging evidence from structural biology and disease pathology with inhibitor profiling and analytical technologies, this review highlights the underexplored therapeutic potential of targeting INMT and underscores its value as a promising target for drug development and therapeutic applications.

## 1. Introduction

Indolethylamine N-methyltransferase (INMT) is a transmethylation enzyme involved in the metabolism of endogenous and exogenous amines, playing a vital role in modulating neurotransmitter activity, maintaining chemical homeostasis, and regulating psychoactive compounds. It catalyzes the methylation of nitrogenous molecules, especially indoles, where it transfers one or more methyl groups from S-adenosyl-L-methionine (SAM) [1]. The enzyme belongs to class 1 of the methyltransferase family and is also referred to as a thioether S-methyltransferase (TEMT); it contributes to neurotransmitter methylation, particularly of indole-based molecules such as tryptamine and serotonin, which influences neuromodulation, perception, and cognitive function [2]. In peripheral tissues, INMT is also involved in detoxification pathways and the methylation of xenobiotic amines, pointing to broader physiological relevance beyond the central nervous system.

This review not only consolidates current knowledge on INMT, but also offers a novel perspective by providing a comprehensive synthesis of INMT’s structure, function, disease associations, inhibitors, and analytical assessment. By highlighting its emerging roles in neuropsychiatry, oncology, and neurotransmitter metabolism, we address a significant gap in the literature where INMT remains underexplored as a therapeutic target and biomarker. We also evaluate the utility of existing analytical tools and inhibitor scaffolds, offering a unique and timely framework for future research and drug development.

### 1.1. History and Discovery of INMT

In 1961, INMT was first discovered, where Axelrod (1961) reported the presence of an enzyme that can methylate indole amines, such as serotonin and tryptamine, to produce N-methylated psychomimetic metabolites [3]. This discovery marked a significant advance in the understanding of endogenous hallucinogens and their metabolic pathways, as it provided the first evidence of an endogenous enzymatic pathway capable of producing psychoactive compounds, such as N-methylated indoleamines. Among these, N,N-dimethyltryptamine (DMT) later emerged as a potent psychedelic molecule with neuromodulatory properties [4]. This exploration suggested that INMT-mediated methylation might influence brain function, mood, and perception [3]. Inspired by this discovery, in the late 90s, Thompson and his colleagues successfully cloned and characterized INMT in both rabbit and human tissues, establishing its presence and preservation across species [5].

### 1.2. Substrates and Physiological Relevance

INMT has a broad substrate specificity and can methylate an array of nitrogenous compounds, such as serotonin, tryptamine, and phenylethylamines, along with other indole-containing compounds [6,7]. Amongst the diverse INMT substrates, tryptamine attracted a special focus owing to its stepwise methylation into N-methyltryptamine (NMT) and ultimately N, N-dimethyltryptamine (DMT) [6]. DMT is an endogenous psychedelic compound with a wide range of actions. In addition to its profound impact on perception, cognition, and mood [8]. DMT interacts with several central nervous system targets, including serotonin receptors, monoamine transporters, and the sigma-1 receptor (S1R), suggesting its implication in numerous psychiatric disorders [9].

### 1.3. INMT in Neuropsychiatric and Neurodegenerative Disorders

Alterations in INMT expression and/or activity have been correlated with neurological conditions and psychotic disorders [6,10]. The involvement of INMT is further supported by DMT’s interaction with the neuromodulator targets such as serotonin receptors, monoamine transporters, and sigma-1 (S1R) receptors, which can exacerbate or contribute to these conditions [7]. Several hypotheses, including but not limited to the transmethylation hypothesis [11], suggested that INMT is a contributing factor in schizophrenia and stress-related psychoses [12,13,14]. Beyond its neuropsychiatric involvement, INMT has also been associated with neurodegenerative diseases such as Alzheimer’s and amyotrophic lateral sclerosis (ALS) [15,16]. The co-localization of INMT with S1R in the motoneurons might explain the possible role of INMT in neuroprotection, signaling function, and disease pathology; nevertheless, the exact molecular mechanism associating INMT with neurodegenerative diseases remains unclear [16].

### 1.4. INMT in Cancer

According to several studies, dysregulated INMT expression has been implicated in the development and progression of several human cancers [17]. For instance, INMT is downregulated in malignancies such as lung and liver cancer, where its low expression correlates with poor prognosis and may contribute to tumor proliferation through impaired detoxification of bioactive amines [18]. Conversely, upregulated INMT expression has been observed in castration-resistant prostate cancer (CRPC), where it may promote chemoresistance via methylation of anticancer agents [19]. These context-specific patterns suggest a complex, tumor-type-dependent role for INMT. A detailed discussion of these findings is provided in Section 3.5.

### 1.5. Enzyme Inhibitors and Regulation

Due to its involvement in various disease conditions, INMT represents a promising, overlooked target that could provide valuable insights into disease pathogenesis. Furthermore, targeting INMT may potentially offer an alternative therapeutic strategy for a range of illnesses, such as cancer, psychiatric disorders, and neurodegenerative diseases. Because of the above-mentioned points, researchers explored INMT inhibitors aiming to modulate its activity and regulate conditions resulting from altered methylation processes.

Both exogenous and endogenous inhibitors of INMT have been previously reported. Regarding endogenous inhibitors, DMT [1], the primary product of INMT, as well as S-adenosyl-L-homocysteine (SAH) [20], a byproduct of the methylation reaction, have been identified as endogenous inhibitors. When their level reaches a certain extent, it provokes a negative feedback mechanism to regulate INMT activity and maintain homeostasis [21].

Synthetic inhibitors, such as propyl dimethyl amino tryptamine (PDAT) [7] and amidine derivatives [22], have been investigated and identified as selective exogenous inhibitors of INMT with potential pharmacological relevance.

### 1.6. Analytical Approaches for INMT Activity Assessment

The measurement of INMT enzyme activity has been reported several times in the literature. It mainly relies on the radiochemical reaction utilizing ^14^C-SAM as a methyl donor to obtain a radioactive tryptamine derivative [1,7,23,24]. Non-radiochemical methods have also been reported utilizing ultra-performance liquid chromatography–mass spectrometry (UPLC-MS/MS) and thin-layer chromatography [25]. Both used classical chromatographic techniques to detect the products (NMT and DMT). Additional methods have been developed and are now commercially available in the form of assay kits. These colorimetric or fluorometric assays utilize 96- and 384-well plates to monitor the enzyme activity [26,27,28,29].

## 2. INMT Characterization and Distribution

**Figure 1:**
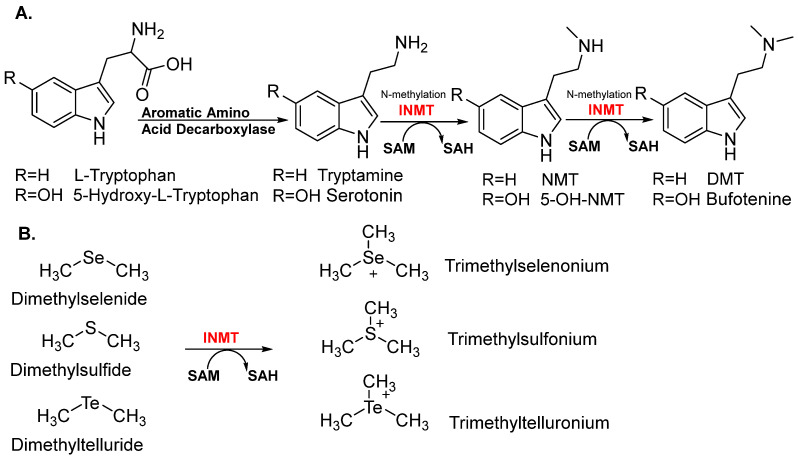
INMT enzyme substrates. (**A**) To synthesize the endogenous DMTs, AADC decarboxylates L-tryptophan and 5-hydroxy-L-tryptophan to tryptamine and serotonin. INMT utilizes SAM (AdoMet) to methylate tryptamine and serotonin to obtain NMT and 5-OH-NMT, and dimethylates to produce DMT and bufotenine. (**B**) INMT catalyzes the methylation of dimethyl derivatives of Se, S, and Te to trimethylselenonium, trimethylsulfonium, and trimethyltelluronium.

The INMT is a transmethylation enzyme that was first isolated from rabbit lungs [3]. It catalyzes the N-methylation of compounds (Figure 1) comprising an indole ring, utilizing SAM as the methyl donor [6]. INMT was primarily recognized for methylating tryptamine into N-methyltryptamine (NMT) and DMT. As a member of the N-methyltransferase enzyme family, INMT can also catalyze the methylation of various indoleamines, including serotonin, 5-methoxytryptamine, phenylethylamine, and other endogenous indole derivatives, although with much lower affinity [7]. It is worth mentioning that INMT catalyzes the methylation of various thioethers and the corresponding compounds containing selenium and tellurium, such as dimethyl telluride and dimethyl selenide [30].

### 2.1. Cloning and Genomic Characterization of INMT

Cloning and expression of INMT cDNA and INMT genes were performed in both rabbit and human models, revealing a 792-base pair open reading frame encoding a 263-amino acid protein [1,5], while sharing about 88% sequence identity with its rabbit counterpart [5]. INMT belongs to a conserved N-methyltransferase family that also includes nicotinamide N-methyltransferase (NNMT) and phenylethanolamine N-methyltransferase (PNMT), which methylate nicotinamide and norepinephrine, respectively. INMT displays 53% sequence identity and 67% similarity with nicotinamide N-methyltransferase (NNMT), while its similarity to PNMT is 38%. Albeit structurally related, INMT is the only member of the family with S-methyltransferase activity, which allows it to transfer a methyl group from SAM to sulfur, selenium, or tellurium atoms of thioethers and related compounds [19]. This enzymatic function is conferred by its distinct spatial arrangement of key catalytic residues within its active site. The human INMT gene is located on chromosome 7p15.2–p15.3 and spans 5471 base pairs [30]. Notably, although the hINMT exon-intron splice junction complies with the “GT-AG” rule, it lacks canonical TATA or CAAT sequences at the 5′-flanking promoter region, suggesting distinct regulatory mechanisms for gene expression [5].

### 2.2. Protein Structure and Active Site of INMT

The hINMT protein consists of 263 amino acids and has an apparent molecular weight of 29.29 kDa. The three-dimensional crystal structure of hINMT was reported by Wu et al. [31] using X-ray crystallography. The crystal structure was deposited in the Protein Data Bank under the PDB code of 2A14, featuring a resolution of 1.70 Å and co-crystallized with SAH. It exhibits a characteristic methyltransferase-like fold, comprising a single polypeptide chain. Additionally, the enzyme possesses signature motifs typical of methyltransferases, including regions crucial for SAM binding and catalytic activity [32].

**Figure 2:**
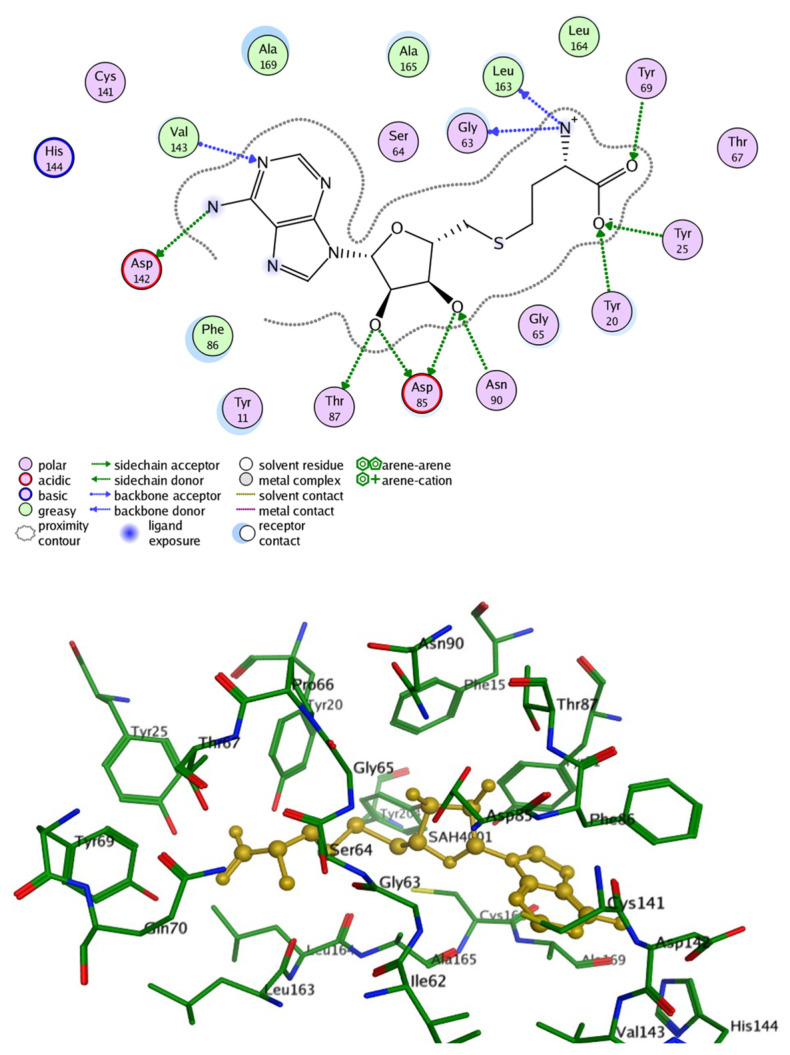
Binding site of hINMT (2A14) with SAH generated by Molecular Operating Environment software (MOE 2010). It displays the molecular interactions between the key amino acids of the binding site and the ligand (SAH).

To highlight the role of INMT’s active-site residues in stabilizing substrate binding and to gain structural insight into substrate recognition, SAH was docked into the crystal structure of the enzyme, which consists of twenty crucial amino acids (PDB ID: 2A14) (Figure 2). The resulting binding poses, as shown in Figure 2, revealed a well-defined network of hydrogen bonding interactions with 10 key residues, stabilizing SAH within the active site. Specifically, the carboxylate moiety of the butanoic acid engaged in hydrogen bonding with Tyr20 and Tyr25, while the carbonyl group formed a hydrogen bond with Tyr69. The positively charged amino group established interactions with Gly63 and Leu163. The hydroxyl groups of the oxolane ring participated in hydrogen bonding with Asp85, Thr87, and Asn90. Furthermore, the amino group of the aminopurine ring interacted with Asp142, and the ring nitrogen at position 1 engaged in hydrogen bonding with Val143.

Despite the limited research on INMT’s binding pocket and key residues involved in substrate interactions, Thompson et al. utilized an experimentally informed computational model approach to characterize the rabbit INMT (rINMT) binding pocket. Subsequently, they confirmed it to be fully conserved in human INMT (hINMT) [33]. The key residues essential for effective interaction with SAM and tryptamine were identified. For SAM docking, it was found that the residue Tyr69 facilitates cation–π interactions, while Gly63, Ser64, Thr87, Glu92, and Glu116 form hydrogen bonds to stabilize the configuration within the binding site, whereas the tryptamine binding region showed hydrogen bonding formed by Lys39 and Gln200 (Arg in hINMT), van der Waals interactions by Glu43, Val138, and Ile202 (Pro in hINMT), and finally π–π interactions with Tyr69.

Another study investigated the structural features of human recombinant INMT that contribute to its methyltransferase activity [30]. Molecular dynamics simulations combined with fragment molecular orbital methods revealed a hydrophobic active site that preferentially binds neutral substrates such as tryptamine and NMT. Mutagenesis studies emphasized the importance of Leu164 in maintaining the substrate access channel, where substitutions with smaller or polar residues (glycine, asparagine) reduced substrate binding affinity and obstructed substrate entry, demonstrating the importance of hydrophobic interactions in stabilizing substrate positioning [30].

### 2.3. Comparative Structural Features Across Species

**Figure 3:**
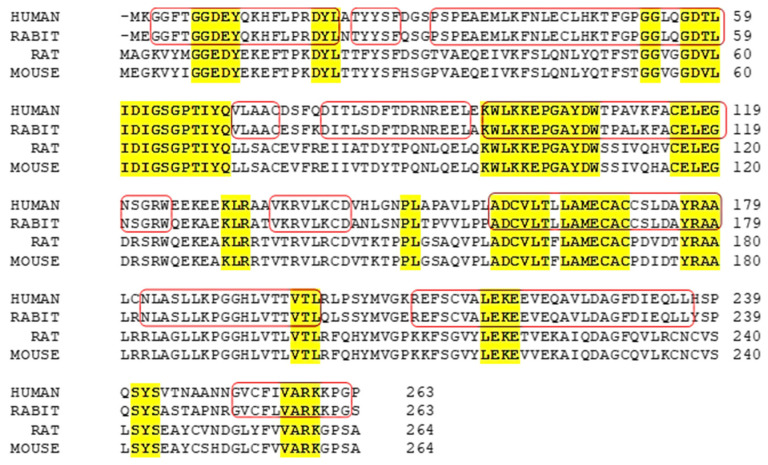
Conserved motifs of INMT in mammals. Sequence alignment of four representative INMT from human, rabbit, rat, and mouse [NP_001186148, NP_001075512, NP_001102492.1, and NP_033375.1]. Conserved motifs across these species are highlighted in yellow, while the identical sequences observed in humans and rabbits are enclosed within a red frame.

Comparative modeling between Bufo INMT (BINMT) and human INMT (hINMT) identified 17 conserved residues within the SAM-binding pocket, supporting conservation of the catalytic region [32]. In BINMT, docking studies showed that indole ethylamines interact with Phe23 via π–π interactions, a functionally analogous role to Tyr69 in hINMT. These interactions appear to align the methyl group of SAM appropriately for efficient methyl transfer, contributing to the enzyme’s specificity. In both species, residues such as Tyr19, Asn199, and Tyr202 were implicated in substrate stabilization, suggesting that aromatic and hydrophobic interactions exerted by these conserved residues contribute to substrate specificity and enzymatic efficiency [32]. Furthermore, the amino acid sequences INMT enzyme across the species revealed that human INMT shares 58% identity with the mouse TEMT, which consists of 264 amino acids [5]. Both enzymes exhibit dual conserved activity of N- and S-methyltransferase. These structural insights reinforce the importance of key residues and conserved interactions in governing substrate recognition and catalytic function across INMT homologs. Conserved motifs of INMT across species are cited in Figure 3.

### 2.4. Tissue Distribution and Functional Activity of INMT

In humans, multiple tissues have shown significant mRNA and protein expression of hINMT [25]. Northern blot analysis of 35 human tissues revealed a 2.7 kb mRNA transcript across different tissues. Expression levels were high in the lung and adrenal gland, in addition to significant levels detected in the spinal cord. Furthermore, INMT is highly colocalized with S1R at the postsynaptic sites of the C-terminal in motor neurons and adrenal tissues [34]. This distribution suggests that INMT is not confined to peripheral metabolic processes but may also play important physiological roles within the central nervous system. Moreover, when expressed in COS-1 cells, hINMT showed prominent methylating activity toward tryptamine, displaying an apparent Km value of 2.92  ±  0.07 mM [5]. Although Thompson et al. [5] reported an absence of hINMT expression in brain tissues. The study relied on Northern blotting, a method with relatively low sensitivity, which may not detect transcripts expressed at low levels or in a highly localized manner [35]. Cozzi et al. proposed that the absence of INMT in the previously mentioned study may be due to regional restriction of expression, inducible transcription, or limitations in the sensitivity of Northern blot analysis [32]. Moreover, recent studies demonstrated the presence of INMT protein in specific brain regions such as the pineal gland and ventral horn of the spinal cord in Rhesus macaques, supporting localized expression [32]. Taken together, these observations suggest that Thompson’s negative findings reflect methodological limitations and the unique spatial regulation of INMT rather than a true absence of expression in the CNS.

Rabbit INMT (rINMT) was the first to be cloned and characterized, paving the way for the identification of hINMT [5]. Rabbit lung tissues displayed the highest INMT expression and the greatest methylating activity toward tryptamine and other indole ethylamines [1]. When expressed in COS-1 cells, rINMT showed a Km of 0.27  ±  0.05 mM for tryptamine, significantly lower than that of the human enzyme [5].

In rodents, an in vivo microdialysis study detected DMT in the dialysate samples obtained from the pineal gland and cortex of rats [36]. The potential role of INMT in the biosynthesis of endogenous tryptamines within the CNS is further supported by the findings from Cozzi et al., where INMT protein expression was identified in the pineal gland of Rhesus macaques [37]. These findings reinforce the hypothesis that INMT contributes to the endogenous production of DMT and related methylated amines in the brain, although its physiological function remains to be fully uncovered.

The widespread expression of INMT in both peripheral and central tissues suggests a potential role in disease. For instance, its localization in the pineal gland links it to autism [38] and supports the transmethylation hypothesis of schizophrenia [11]. Additionally, INMT is highly expressed in the lung, where its dysregulation has been implicated in certain lung cancers [39]. Moreover, co-localization of INMT with the sigma-1 receptor (S1R) in motor neurons suggests a possible involvement in neurodegenerative diseases such as Alzheimer’s disease (AD) [14], amyotrophic lateral sclerosis (ALS), and other motor neuron disorders [40].

Collectively, the diverse tissue distribution of INMT provides a mechanistic rationale for its emerging associations with neuropsychiatric disorders, cancer, and neurodegeneration, which will be discussed in the following section.

## 3. INMT and Related Disorders

**Figure 4:**
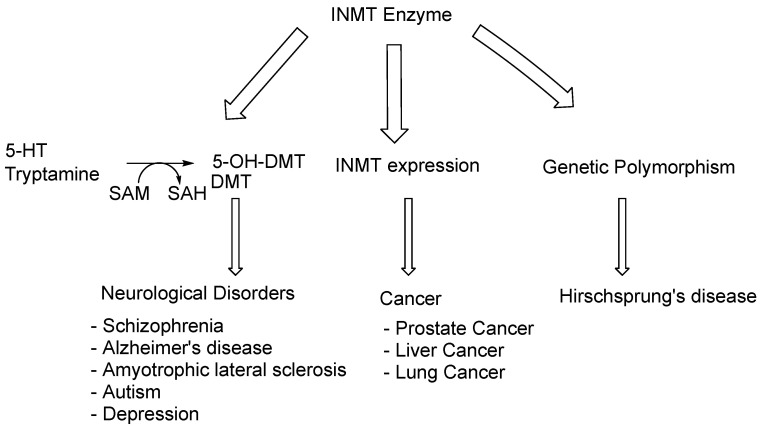
Diseases and mechanisms associated with INMT.

Several studies discussed the role of INMT activity and its metabolite DMT in some medical disorders, including schizophrenia and stress-related psychoses [12,13,14]. Other studies revealed the relationship between INMT expression and prostate cancer [17]. Two distinct hypotheses have been proposed for INMT’s contribution, such as its role in detoxifying the volatile selenium compounds. It was found that the activity interferes with the selenium-based anticancer drugs, potentially leading to the development of castration-resistant prostate cancer [17]. Interestingly, it has also been reported that INMT downregulation may contribute to lung and prostate cancer [18,19]. However, the INMT’s exact role and the molecular mechanism in cancer and other disorders remain unclear. The INMT-associated disease and mechanisms are summarized in Figure 4.

### 3.1. Schizophrenia

Schizophrenia is a complex and heterogeneous mental disorder, characterized by a combination of signs and symptoms including cognitive, behavioral, and emotional dysfunctions. Approximately 1% of the worldwide population suffers from schizophrenia, and it represents a median lifetime morbid risk of less than 1% [41,42]. One of its main characteristics is cognitive impairment, which significantly contributes to the functional disabilities associated with the disorder [43]. The onset of symptoms usually occurs in late adolescence or early adulthood. Hallucinations, paranoid delusions, and disordered thinking or behavior are examples of positive symptoms. Negative symptoms include low motivation, low energy, and disregard for personal hygiene [44]. Despite the extensive research on schizophrenia, the underlying pathophysiology remains unknown. Additionally, no reliable diagnostic neuropathology or sensitive and specific biomarkers have yet been identified [41]. However, several hypotheses have been proposed to explain its pathoetiology [43,45]. Some of these hypotheses suggest that INMT may play a role in schizophrenia, offering a potential target to overcome its devastating effects.

The transmethylation hypothesis [11] originally proposed that schizophrenia may arise from abnormal metabolism in the adrenal glands, where excessive methylation of catecholamines such as adrenaline produces mescaline-like compounds with hallucinogenic properties. These compounds possess a psychedelic effect, and it is believed that they can contribute to the clinical symptoms associated with schizophrenia. Although this process involves a different enzymatic pathway, the hypothesis has since been extended to include endogenous tryptamines. In this context, DMT, an endogenous compound, is synthesized via the N-methylation of dietary tryptamine by the INMT enzyme [4].

Dysregulation in the enzymatic activity of INMT results in elevated levels of methylated indole alkylamines, including DMT and its psychoactive analogs, such as 5-hydroxy-DMT (bufotenine) and 5-methoxy-DMT, in individuals with schizophrenia [4,13]. Elevated levels of DMT and its analogs have been linked to the positive symptoms of psychosis in psychiatric patients [46]. Furthermore, patients suffering from acute paranoid schizophrenia display a higher level of methylated indole alkylamines compared to healthy controls [47]. Notably, INMT is highly expressed in the adrenal glands, supporting the early hypothesis that a metabolic disorder involving these glands could play a role in the pathophysiology of schizophrenia [5]. Stress has also been hypothesized as a contributing factor in schizophrenia, particularly in the development of the positive symptoms associated with the disorder [48]. Furthermore, the intensity of psychotic symptoms is exacerbated in response to stressful conditions [49]. Notably, stress has been associated with increased levels of DMT, suggesting a possible link between stress and increased tryptamine metabolism [11]. Studies show that DMT precursors increase in response to stress. For instance, animal models demonstrate that stress results in an increase in adrenal tryptamine [50], as well as higher concentrations of tryptophan and tryptamine in the brain [51].

DMT has been proposed to act as a neurotransmitter, with serotonin transporters (SERT) on the neuronal plasma membrane facilitating its uptake. Subsequently, DMT is stored in synaptic vesicles by vesicular monoamine transporter 2 (VMAT2) for release at receptor sites [52]. Increased densities of both serotonin transporters (SERT) and vesicular monoamine transporter 2 (VMAT2) have been observed in patients with schizophrenia [53,54]. Furthermore, a study conducted by Tillinger et al. demonstrated that vesicular monoamine transporter 2 (VMAT2) mRNA expression rises in response to stress [55] further highlighting the role of DMT and stress in the positive symptoms of psychosis. Additionally, there have been case reports of acute psychosis in individuals with no prior psychiatric history following DMT consumption [12]. Additionally, stress is believed to reduce the activity of monoamine oxidases (MAOs), the enzymes responsible for breaking down DMT and other monoamines, leading to elevated DMT levels [56]. It has been observed that individuals with psychotic disorders exhibit decreased activity of monoamine oxidase (MAO) and often experience perceptual abnormalities [13].

Previous studies demonstrated an increase in INMT activity in patients suffering from schizophrenia. A study conducted by Wyatt et al. measured the activity of INMT in platelets of schizophrenic patients in comparison to normal subjects, using NMT and radiolabeled ^14^C-SAM as substrates. Results deem that the mean enzymatic activity in chronic schizophrenic patients (n = 30) was significantly higher (*p* < 0.001) compared to the normal group (n = 49). Similarly, the INMT activity was higher in acute schizophrenic patients (n = 5) compared to non-schizophrenic participants (*p* < 0.01) [6]. Another comparative study showed that serum INMT activity was significantly higher in paranoid schizophrenic individuals compared to other types and was positively correlated with the severity of delusions in all psychiatric and schizophrenic patients. This suggests that INMT may play a role in the biochemical processes underlying delusional symptoms [10].

INMT can also catalyze the conversion of 5-HT into psychomimetic 5-OH-DMT [2]. Emanuele et al. measured the level of 5-OH-DMT in the urine of young adults with schizophrenia (n = 15) and healthy control subjects (n = 18). It was found that the 5-OH-DMT levels were significantly higher in patients with schizophrenia (4.39 ± 0.43 µg/L, *p* < 0.001) compared with controls (1.53 ± 0.30 µg/L) [14]. Another psychomimetic indolamine synthesized by INMT is 5-MeO-DMT, formed by methylation of 5-MeO-tryptamine. It was evinced that 5-MeO-DMT can alter the firing pattern of medial prefrontal cortex pyramidal neurons in rodents, as well as decrease cortically generated slow oscillations that constitute the synchronization of global cortical networks [57], suggesting the involvement of 5-MeO-DMT in schizophrenia, as the disruption of cortical synchronization is a common feature observed in this condition [58].

It is important to note that many of the early studies investigating DMT levels in psychiatric patients had limitations. These included outdated analytical methods, small sample sizes, and the rapid metabolism of DMT by liver monoamine oxidases (MAOs), which affected the accuracy of the measurements taken from body fluids. Barker et al. reviewed 69 published studies from 1955 to 2010. The review highlighted the shortcomings of existing data and argued that direct measurement of DMT levels in bodily fluids may not be optimal to reflect its role in schizophrenia [59]. Therefore, assessment of DMT metabolites or, preferably, INMT activity, might offer a more promising alternative to the current limitations and a clearer understanding of INMT’s involvement in schizophrenia [2]. In addition to the transmethylation hypothesis, another hypothesis suggesting INMT might be involved in schizophrenia is the dopamine hypothesis. The hypothesis posits that dopaminergic hyperactivity is linked to the psychotic symptoms observed in schizophrenia. This hyperactivity comprises the positive symptoms associated with schizophrenia and is a result of increased sensitivity and density of dopamine D2 receptors in specific brain regions [60]. A recent update on the dopamine hypothesis implies that schizophrenia is characterized by dual dysregulation of the dopaminergic system. The decline in dopamine activity in the prefrontal cortex contributes to the negative symptoms, whereas excessive dopamine activity in the mesolimbic systems constitutes the positive symptoms [61]. Several animal studies suggest that DMT acts as an indirect modulator of the dopaminergic system. For example, DMT has been shown to increase dopamine release from synaptic stores [62] and stimulate its striatal synthesis in rats [63]. These observations are further supported by the rise in 3-methoxytyramine, a major dopamine metabolite, following acute and chronic administration of DMT [64]. These findings are in correspondence with the study conducted by Glynos et al., which demonstrated that intravenous administration of DMT resulted in a dose-dependent elevation of dopamine and serotonin levels in both the medial prefrontal and somatosensory cortices of rats [65]. Due to the influence of DMT on dopamine regulation, targeting INMT may be a promising strategy for modulating dopaminergic activity in schizophrenia.

Another potential implication of DMT in schizophrenia pathophysiology is its interaction with S1Rs. Sigma-1 receptors are colocalized with INMT at the postsynaptic sites of the C-terminal in motor neurons and adrenal tissues [34]. This proximity suggests that DMT may be synthesized locally to activate S1Rs, highlighting the possibility of a direct molecular interaction between them [34]. Fontanilla et al. identified DMT as an endogenous S1R agonist. Additionally, this interaction between DMT and S1R might be responsible for the hypermobility observed in animal models [15], a well-established behavioral feature associated with schizophrenia [66]. The study also revealed that hypermobility was not observed in S1R knockout mice and rodents treated with the S1R receptor antagonist haloperidol [67], emphasizing the role of S1R and DMT in regulating motor activity and behaviors associated with psychosis. In addition to its role in hypermobility, S1R activation by DMT has been suggested to mediate hallucinogenic effects by acting on various voltage-sensitive ion channels [68]. This indicates that S1R activation by DMT may play a role in the sensory and perceptual distortions seen in hallucinatory states, albeit the precise connection to schizophrenia remains unclear.

### 3.2. Alzheimer’s Disease (AD)

In Alzheimer’s disease (AD), key pathological features, such as amyloid-beta (Aβ) plaque accumulation [69,70], mitochondrial dysfunction, impaired ER-mitochondria crosstalk [71], and decreased S1R activity, contribute to the cognitive impairment and neurodegeneration observed in pathology [72]. A recent study explored the potential of DMT, being an endogenous agonist of S1R, to ameliorate these pathological hallmarks in a transgenic (TG) AD mouse model (3×TG-AD) [16]. The study revealed that chronic administration of DMT (2 mg/kg) for 3 weeks significantly improved cognitive deficits in transgenic mice, as evidenced by performance in the water maze test. Additionally, it substantially reduced Aβ deposits in the hippocampus and prefrontal cortex. DMT also restored the reduced S1R levels observed in these transgenic mice while improving neuronal ER-mitochondria crosstalk [14]. Highlighting the potential anti-Alzheimer’s effects of DMT through S1R activation. Trace amines, such as DMT, have the potential to bind with high affinity and activate trace amine-associated receptor 1 (TAAR1) receptors [35], which may contribute to Alzheimer’s disease (AD) pathology [73]. Dhakal and Macreadie (2021) suggested that TAAR1 activation could exacerbate N-methyl-D-aspartate (NMDA) receptor-mediated excitotoxicity, a key factor in AD progression [73]. Blocking TAAR1 activity may help restore calcium and sodium balance in neuronal cells, thereby improving calcium homeostasis and reducing mitochondrial reactive oxygen species (ROS) production [73]. As a result, inhibiting INMT could represent a promising therapeutic approach to alleviating AD pathology. Zhong et al. reported that INMT can also metabolize Bis (7)-tacrine, a multi-target anti-Alzheimer’s and antiproliferative agent, detailing that the activity of Bis (7)-tacrine showed a 549-fold improvement in INMT-knockdown models compared to controls [19].

### 3.3. Amyotrophic Lateral Sclerosis (ALS)

Amyotrophic lateral sclerosis (ALS) is a progressive neurodegenerative disease characterized by the degeneration of spinal cord motor neurons, leading to muscle weakness, paralysis, and ultimately, respiratory failure [74]. The S1Rs are highly expressed in motor neurons of the spinal cord, and are particularly enriched in C-terminals, which are uniquely structured cholinergic postsynaptic sites [75]. They regulate motor neuron function by modulating calcium signaling, stabilizing mitochondria, and controlling neuronal excitability, thereby reducing the risk of motor neuron hyperactivity and oxidative damage associated with ALS [76]. Their neuroprotective role in ALS is demonstrated by studies showing faster disease onset and decreased longevity in S1R knockout mice [77] and improved prognosis in ALS models treated with S1R ligands [78]. As previously mentioned, INMT colocalizes with S1Rs in motor neurons [34] and can contribute to protection against ALS pathology through two main functions. First, it produces DMT, a well-known endogenous S1R agonist [15], which may help sustain the protective effects of S1R activation. Second, INMT plays an important role in the methylation and detoxification of toxic sulfur and selenium derivatives, which are known contributors to ALS [40]. Considering the potential role of INMT in ALS, developing small molecules that modulate INMT activity could provide new therapeutic avenues to delay the disease progression and improve therapeutic outcomes.

### 3.4. Autism

Although the neurophysiological causes of autism remain obscure, one of the main contributing factors is the dysfunction of the pineal gland [38] and the consequent deficiency in its primary hormone, melatonin [79]. This is supported by the findings that individuals with autism spectrum disorders (ASD) display significantly lower levels of melatonin and a higher prevalence of sleeping disorders [80]. Melatonin levels are approximately less than half the average values in 65% of individuals suffering from ASD [80]. Furthermore, in developing children, the prevalence of sleep disorders in ASD is notably higher compared to normal children, affecting 50–80% compared to 9–50% [81]. Moreover, the administration of exogenous melatonin has been proven to improve the onset as well as the sleep duration [82].

It has been proposed that the dysfunction of the pineal gland increases the INMT activity, diverting tryptophan metabolism, the precursor of both melatonin and DMT, towards DMT synthesis at the expense of melatonin, leading to the declined level of melatonin commonly observed in ASD [79]. Furthermore, the pineal gland is thought to be the main source of DMT in the brain [36], and dysfunction of the gland is believed to affect DMT metabolism in addition to its function in melatonin production [83].

A common feature of ASD is the abnormal neuroplasticity, which is represented by cortical overgrowth and dendritic spine dysgenesis [84]. According to a study conducted by Ly et al., DMT affects neuritogenesis, spinogenesis, and synaptogenesis in animal models by activating 5-Hydroxytryptamine 2A (5-HT2AR), tropomyosin receptor kinase B (TrkB), and mTOR signaling pathways [83]. These findings suggest that DMT hyperactivity resulting from pineal gland dysfunction may contribute to the aberrant neuroplasticity and neural connectivity deficits observed in individuals with ASD [79]. This is consistent with another study that reported significantly elevated urine levels of bufotenine (5-OH-DMT) in patients with schizophrenia (4.39 ± 0.43 µg/L, *p* < 0.001) and ASD subjects (3.30 ± 0.49 µg/L, *p* < 0.05) compared to controls (1.53 ± 0.30 µg/L) [14].

### 3.5. Cancer

#### 3.5.1. Castration-Resistant Prostate Cancer (CRPC)

Prostate cancer is one of the most common malignancies and is the second leading cause of cancer-related death in men [85]. Although patients initially respond to androgen deprivation, ultimately, all cancers become castration resistant. Treatment for CRPC has a very poor prognosis and still represents a major clinical challenge [86]. Zhong et al. investigated the role of INMT in CRPC. The results show that the overexpression of INMT resulted in a poor prognosis for clinical prostate cancer. Contrastingly, INMT knockdown cell lines prevented CRPC growth and development, and notably enhanced the antineoplastic effect of exogenous anticancer agents [17]. One proposed mechanism is that INMT promotes resistance by methylating exogenous anticancer metabolites such as methylseleninic acid (MSA) and methylselenocysteine (MSC) [19]. These compounds act as prodrugs for methylselenol, the active anticancer agent. However, when monomethylated selenium species are further methylated by INMT into dimethylated or trimethylated forms, their anticancer efficacy is reduced due to increased metabolic inactivation and rapid excretion [19].

#### 3.5.2. Lung Cancer

To determine the molecular changes in HIV-associated lung cancer and detect related biomarkers. Biomarker screening using Affymetrix microarrays, ingenuity pathway analysis (IPA), and gene expression profiles were analyzed in 59 patients with HIV-associated lung cancer [87]. The results demonstrate that INMT mRNA expression was downregulated in HIV lung cancer patients. Similarly, Kopantzev et al. reported INMT downregulation in non-small cell lung cancer (NSCLC) compared to normal tissues using polymerase chain reaction (PCR) and microarray analyses [39]. These findings are consistent with the study by Zhou et al., which investigated the clinical significance of INMT in NSCLC, particularly in lung adenocarcinoma (LUAD). The findings confirm results from earlier studies indicating that patients with NSCLC display a significant reduction in INMT expression compared to normal cells (*p* < 0.001). Additionally, Kaplan–Meier survival analysis revealed a correlation between the decline in INMT expression and poor clinical outcomes in lung adenocarcinoma (LUAD) patients. The difference in the overall survival between low and high INMT expression groups was the most pronounced among NSCLC patients. Interestingly, despite the poor prognosis, patients with low INMT expressions showed better response to immunotherapy, such as anti-PD-1 treatment. One proposed mechanism linking low INMT expression to prognosis is that the INMT enzyme has a role in detoxifying various cancer cells, particularly by methylating biogenic monoamines possessing mitogenic activity, such as serotonin, tryptamine, and tyramine. It was found that serotonin might promote liver tumor growth and be involved in the development of several cancers. Reduced INMT activity could lead to the accumulation of serotonin in tumors, thereby promoting cell proliferation [17].

#### 3.5.3. Hepatocellular Carcinoma

Studies have shown that the expression of INMT is downregulated in hepatocellular carcinoma. Transcriptome analysis of hepatocellular carcinoma rat models revealed reduced expression of the INMT gene and its encoding protein. Furthermore, survival analyses evinced that INMT downregulation is linked to poor prognosis in humans [88]. Likewise, another study demonstrated that INMT knockdown significantly increased liver cancer cell proliferation and colony establishment, suggesting that INMT might act as a tumor-suppressing agent in hepatocellular carcinoma [89].

#### 3.5.4. Other Cancer Types

INMT downregulation has been observed in other types of cancer, such as meningioma, prostate cancer, and uterine corpus endometrial carcinoma, and is associated with poor overall survival [17]. INMT downregulation appears to be a common feature in multiple cancers. Results from different databases, such as the Tumor Immunity Estimation Resource and The Cancer Genome Atlas databases, have shown reduced INMT expression in several cancers, including cancer of the bladder, breast, cervix, colon, head and neck, esophagus, kidney, lung, prostate, skin, thyroid, and uterus [17,90]. The observed pattern suggested that INMT plays a role in cancer development and may impact tumor progression, cell proliferation, and the tumor microenvironment. This indicates the potential for INMT to serve as a valuable diagnostic and prognostic biomarker for various cancers.

### 3.6. Hirschsprung’s Disease (HSCR)

Hirschsprung’s disease (HSCR) is a congenital disorder that affects approximately 0.02% of newborns. It is characterized by the loss of ganglionic cells either in a segment or the entirety of the colon, leading to intestinal obstruction and other gastrointestinal-related complications [91]. Kim et al. analyzed 15 INMT single-nucleotide polymorphisms (SNPs) to explore the potential association of INMT with HSCR in 187 HSCR patients. It was shown that a nonsynonymous single-nucleotide polymorphism (SNP) rs77743549 of INMT, which results in the substitution of histidine at position 46 with proline (His46Pro), was strongly associated with a higher risk of developing HSCR with an odds ratio of 1.77 and a corrected *p*-value of 0.002 [92]. The mutation has been shown to influence the length of aganglionosis during enteric nervous system (ENS) development as a result of replacing a polar histidine with a rigid proline in the N-terminal region, disrupting the local folding or protein stability [92].

These findings indicate that INMT might play a role in HSCR pathogenesis and could potentially affect neural crest cell development and the severity of HSCR. Further studies are required to determine its exact function and assess its potential to be used as a diagnostic biomarker.

Collectively, the disorders associated with INMT reflect the diverse yet mechanistically convergent roles of the enzyme in human pathology. Among them, schizophrenia garnered the most research attention, likely due to the compelling evidence linking INMT activity and DMT production to psychotic symptoms and serotonergic dysregulation [46]. In contrast, conditions such as Alzheimer’s disease and ALS involve INMT’s interaction with sigma-1 receptors and neuroprotective signaling, highlighting its relevance beyond neurotransmitter methylation [78]. In cancer, INMT exhibits dual roles, either promoting drug resistance (as in prostate cancer) [19] or acting as a tumor suppressor (as in hepatocellular carcinoma and NSCLC) [89], suggesting a context-specific effect tied to its methylation capacity. Meanwhile, genetic polymorphisms in INMT, such as His46Pro, underline the enzyme’s developmental significance, as seen in Hirschsprung’s disease. While the level of mechanistic clarity varies, these conditions collectively support the notion that INMT is a multifaceted enzyme whose dysregulation contributes to pathogenesis. Further comparative studies are warranted to determine whether INMT represents a viable diagnostic or therapeutic target across these distinct disease categories.

## 4. INMT Inhibitors

**Figure 5:**
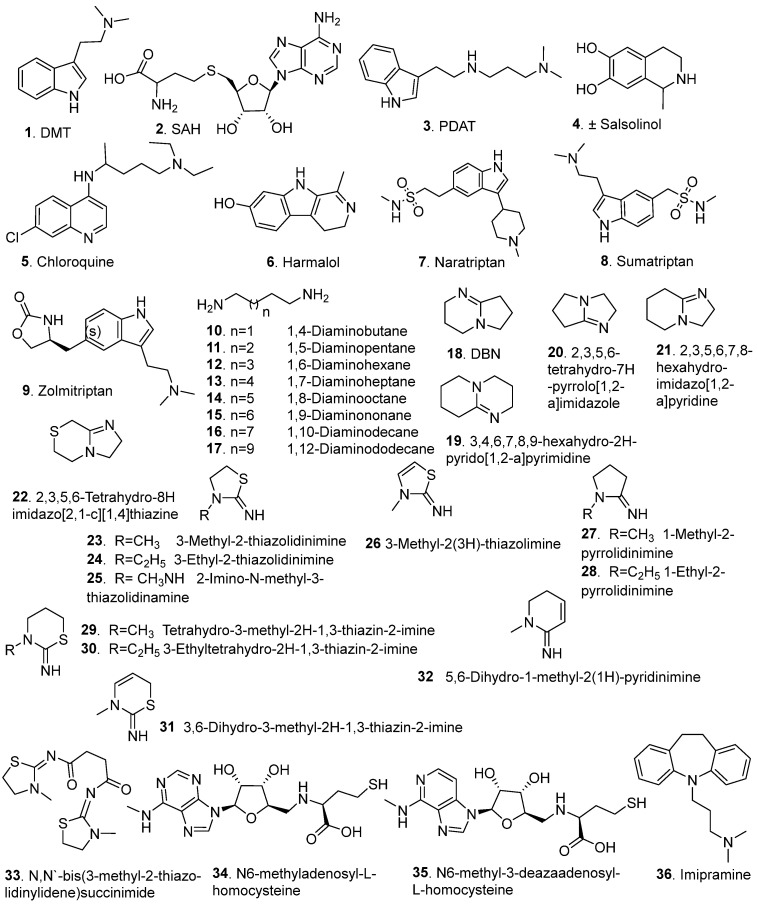
Chemical structures of the INMT inhibitors.

Several inhibitors (Figure 5) have been identified for the INMT enzyme, broadly classified into two categories: endogenous or natural inhibitors and synthetic inhibitors. Figure 5 illustrates the structural diversity of these molecules, ranging from simple endogenous metabolites, such as DMT and SAH, to more complex synthetic scaffolds, including indole derivatives, quinolines, alkylamines, amidines, and homocysteine derivates. Their inhibitory activity was assessed using various enzymatic assays (Table 1). The chemical class, selectivity, potency, and inhibition mechanism are summarized in Table 2.

### 4.1. Endogenous Inhibitors

Endogenous inhibitors of INMT include its metabolic products, which participate in a feedback mechanism to regulate enzymatic activity. DMT **1**, a primary reaction product of INMT, has been shown to inhibit rabbit lung INMT activity by 97% at 1 mM, with an IC_50_ of 67 µM, as quantified using a radiometric assay (M1, Table 1) [1]. Kinetic analysis confirmed competitive inhibition, with computational modeling and docking studies demonstrating the presence of a non-active binding site at the N-terminus by which DMT acts as a non-competitive inhibitor [7].

Similarly, SAH **2**, a byproduct of SAM-dependent methylation, demonstrated potent competitive inhibition of INMT with a Ki of 10 µM when assessed fluorometrically (M2, Table 1). SAH exhibits higher binding affinity than SAM, underscoring its regulatory potency [20]. Both DMT and SAH contribute to a negative feedback loop that regulates INMT activity by preventing excessive methylation and maintaining metabolic balance [21].

### 4.2. Synthetic Inhibitors

Synthetic inhibitors of INMT span several structurally diverse classes, providing insights into structure–activity relationships (SAR) and mechanisms of inhibition. Propyl dimethyl amino tryptamine (PDAT) **3** has been identified as a selective non-competitive inhibitor of rabbit lung INMT [7]. Its inhibitory activity was assessed in an INMT radiometric enzymatic assay (M1, Table 1) and exhibited a Ki value of 84 μM. The selectivity of PDAT 3 is demonstrated by the lack of inhibition of hPNMT or hNNMT. Kinetic studies showed that it exerts pure noncompetitive inhibition by binding to a non-active site near the N-terminus [7].

Thompson and Weinshilboum (1998) explored a series of potential inhibitors of rabbit lung INMT using the assay described above (M1, Table 1) [1]. Amongst the tested compounds, (±)-salsolinol **4**, chloroquine **5**, and harmalol **6** showed the most prominent inhibition, displaying INMT activity percentage, relative to 1 mM tryptamine of 3, 2, and 11%, respectively. These compounds share aromatic or heterocyclic structures that resemble INMT’s substrates, enabling them to interfere with substrate binding. Triptan anti-migraine drugs were also hypothesized to exert an INMT inhibitory activity, which is attributed to their structural similarity with DMT, being indolethylamines. INMT inhibition assays showed IC_50_ values of 147, 370, and 483 μM for naratriptan **7**, sumatriptan **8**, and zolmitriptan **9**, respectively. Moreover, tricyclic antidepressants, such as imipramine 36, displayed an inhibitory activity for INMT with an IC_50_ of 166 μM, due to π–π interactions or hydrogen bonding enabled by its planar tricyclic ring system and dimethylamino substitution [1].

Aliphatic diamines **10–17** represent a structurally simple yet informative class of INMT inhibitors, where inhibitory potency is closely linked to chain length. Porta et al. demonstrated that INMT inhibition increased with alkyl chain length, peaking at eight carbon atoms. This trend highlights an optimal spatial configuration for interaction with the INMT active site [23]. 1,7-diaminoheptane **13** and 1,8-diaminooctane **14** emerged as the most potent members of the series, exhibiting 84.9% and 94.3% inhibition at 10 mM, respectively. Notably, further elongation of the carbon chain beyond eight atoms resulted in a significant decline in activity, suggesting steric hindrance or loss of favorable conformational fit. The K_i_ values indicated that these inhibitors bind with a similar affinity to tryptamine, displaying a competitive inhibition mechanism [23].

The inhibitory activity of 2,3,4,6,7,8-Hexahydropyrrolo[l,2-a] pyrimidine (DBN) **18** was evaluated by Mandel (1976) using an in vivo radiometric assay (M3, Table 1) [93]. DBN **18** displayed significant INMT-inhibitory activity across multiple sources, including rabbit liver and brain, rhesus monkey lung, and human lung tissue. However, it displayed diminished activity against INMT prepared from human liver and rats, suggesting that DMT might be produced through an alternative pathway in rats. This observation aligns with the recent findings indicating that INMT is not necessary for tryptamine methylation or the biosynthesis of NMT and DMT in rat brain and lung tissues [25]. At 200 μM, DBN **18** induced almost complete inhibition of enzyme activity. Mechanistically, DBN 18 acts as a reversible, non-competitive inhibitor of INMT, exhibiting a K_i_ of ~20 μM [93]. The bicyclic amidine scaffold appears to confer both potency and selectivity, as DBN **18** showed no inhibitory effects against other structurally related methyltransferases (phenethanolamine- and imidazole-N-methyltransferases), reinforcing its specificity for INMT [93]. These findings highlight the importance of the fused bicyclic ring system in achieving non-competitive inhibition and selectivity, setting the foundation for subsequent structural optimization of this pharmacophore.

Following the discovery of DBN **18** [93], Rokach et al. [22] explored a wide array of mono-, bi-, and tricyclic amidine analogues as potential INMT inhibitors. Among the bicyclic derivatives tested, four compounds, **19** (IC_50_ = 0.7 μM), **20** (IC_50_ = 0.04 μM), **21** (IC_50_ = 0.17 μM), and **22** (IC_50_ = 0.5 μM), demonstrated superior potency compared to DBN **18** (IC_50_ = 1.67 μM). SAR analysis revealed that various key trends, including smaller ring systems, conferred greater potency, as enlarging the bicyclic core diminished activity, substitution with bulky or aromatic moieties significantly reduced inhibitory efficacy, suggesting that spatial constraints at the binding site limit accommodation of large side chains, and methylation on the five-membered ring slightly attenuated activity, while modifications to the six-membered ring and formation of quaternary amines caused pronounced loss of potency. These findings underscore the importance of scaffold rigidity and steric compatibility with the INMT binding pocket in achieving high-affinity inhibition [22].

Regarding monocyclic amidines (**23–32**), compounds **23**, **24**, and **32** exhibited IC_50_ values below 1 μM, while compounds **25–31** showed slightly lower potency with IC_50_ values ranging from 1.5 to 2 μM (Table 2). SAR insights revealed that the presence of a methyl group on the ring nitrogen and a free exocyclic imine nitrogen significantly enhanced inhibitory activity. Conversely, removal of the ring nitrogen or extension of the alkyl chain beyond an ethyl group reduced potency. Substituent size on the exocyclic imine was inversely correlated with activity; bulkier groups diminished inhibition, while methyl or ethyl groups were optimal. Remarkably, seven out of the ten most active inhibitors contained a sulfur atom adjacent to the exocyclic imine, suggesting a favorable role for sulfur in binding. In contrast, tricyclic amidine analogues failed to show appreciable inhibition, indicating that increased molecular rigidity or steric hindrance impairs binding to the INMT active site [22].

Following the identification of 2-imino-3-methylthiazolidine **23** as a potent INMT inhibitor [22], Rokach et al. pursued the design of derivatives aimed at enhancing in vivo activity and pharmacodynamic properties [24]. N, N′-bis(3-methyl-2-thiazolidinylidene) succinimide **33** incorporated a succinimide linker between two thiazolidine moieties, effectively increasing molecular stability and systemic exposure. Although compound **33** did not exhibit inhibitory activity in vitro, it demonstrated marked in vivo efficacy following oral or intravenous administration in rabbits. This profile suggested that **33** functions as a prodrug of compound **23**, a hypothesis confirmed through a radiometric metabolic study using ^14^C-labeled **33**, which showed conversion back to the active parent compound in whole animals and isolated liver tissue. It illustrates that the prodrug design can overcome metabolic limitations and enhance therapeutic potential without altering the core pharmacophore [24].

Inspired by the inhibitory potential of SAH **2** [20], Borchardt et al., evaluated a series of synthetic SAH analogues to define structural features essential for selective INMT inhibition [94]. The enzyme displayed strict structural requirements for effective binding. It has been shown that the L-configuration of the amino acid is essential for activity, in addition to the presence of both the terminal amino and carboxy groups. Furthermore, a three-carbon spacer between the thiol and the terminal functional groups yields the most significant inhibition. The adenine moiety was also crucial for activity; replacing it with other groups, such as pyrimidine or purine bases, abolished the activity. The 6-amino group is essential for binding, as dimethylation resulted in complete loss of inhibition. The ribose sugar also contributed significantly to INMT selectivity, particularly the 2′-OH group, which was critical for effective enzyme interaction. Among the analogues, N6-methyladenosyl-L-homocysteine **34** and N6-methyl-3-deazaadenosyl-L-homocysteine **35** emerged as the most potent and selective INMT inhibitors, showing minimal inhibitory effects on other methyl transferases, such as catechol O-methyltransferase (COMT), phenylethanolamine N-methyltransferase (PNMT), histamine N-methyltransferase (HMT), and hydroxyindole O-methyltransferase (HIOMT). The structure–activity relationship (SAR) of SAH analogues highlights the specific structural significance of these compounds to achieve optimal potency and selectivity [94].

The pharmacological relevance of INMT inhibitors is underscored by their potential role in modulating pathologies where INMT level and/or activity is upregulated, particularly for psychotic disorders [10], and various malignancies [17,18]. Several inhibitors, such as PDAT **3**, DBN **18**, and synthetic SAH analogues **34** and **35,** demonstrated high selectivity for INMT over related methyltransferases such as PNMT, NNMT, and COMT, reducing the risk of off-target methylation events that could compromise neurological or systemic function [7,22,94]. Furthermore, repurposed clinical drugs with INMT-inhibitory activity, such as chloroquine **5** and imipramine **36**, possess established toxicological profiles, enabling more rapid translational assessment. It is worth mentioning that most of the current literature focuses on INMT inhibition due to its implications in cancer progression and psychosis-related pathways. However, considering that INMT downregulation has been associated with adverse outcomes in certain cancers and neurodegenerative conditions, future studies are warranted to explore whether enhancing INMT activity, either through SAM supplementation or novel small-molecule activators, may offer therapeutic value.

In summary, the inhibition of INMT is mediated by a structurally diverse set of compounds, comprising endogenous regulators, such as DMT and SAH, as well as synthetic scaffolds, including tryptamine analogs, aliphatic diamines, amidines, and SAH derivatives. These inhibitors exhibit varying potencies and inhibition mechanisms. Selectivity toward INMT related to methyltransferases has been demonstrated for key compounds such as PDAT **3**, DBN **18**, and SAH analogues. Collectively, these inhibitors offer valuable tools for probing the physiological roles of INMT and provide promising leads for therapeutic development targeting methylation-linked disorders.

## 5. Measurement of INMT Enzyme Activity by Discontinuous Assays

**Figure 6:**
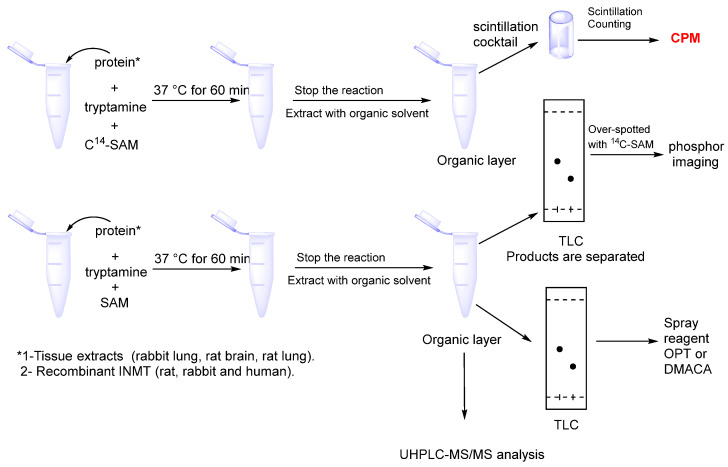
Schematic representation of the radiometric and non-radiometric assays. The reaction mixture includes the INMT enzyme, substrate (tryptamine), and methyl donor (^14^C-SAM). The reaction products in radiometric assay are extracted by organic solvent and detected by TLC/phosphor imaging or measured by scintillation counting. For the non-radiometric assay, the product detection could be achieved by TLC in conjunction with spray reagents such as OPT and DMACA, or by utilizing UHPLC-MS/MS.

Detecting the INMT’s activity has been documented several times in the literature. For screening assays, the INMT enzyme is typically extracted from the tissues of rabbit lung, rat lung, and brain, or sourced from the commercially available recombinant INMT. The most commonly used discontinuous assays are illustrated in Figure 6.

### 5.1. Radiometric Assay

The assay relies on the transmethylation of a labeled methyl group from ^14^C-SAM to the substrate (indoleamine), and the resulting radioactive product is detected using scintillation counting. The second detection method involves using thin-layer chromatography (TLC) combined with an appropriate visualizing technique such as phosphor imaging. The highly sensitive radiometric assay could detect a very low enzyme activity, but it does not provide enough information on product identity [25].

### 5.2. TLC and Spray Reagent Method

The reaction product (indoleamine derivatives) could be detected using spray reagents such as O-phthalaldehyde (OPT) or p-dimethylamino cinnamaldehyde (DMACA) spray reagents. The non-radiometric assay with TLC detection is a fast method to visualize the reaction product, but it lacks the resolution [20,95].

### 5.3. UHPLC-MS/MS Analysis Method

The INMT assay requires a method that provides a high degree of sensitivity and resolution for product identification. This can be accomplished by ultra-high-performance liquid chromatography-tandem mass spectrometry. Glynos et al. developed a UHPLC-MS/MS method to measure the INMT activity. The precursor, reaction product ions, and spiked deuterated internal standards were separated, identified, and quantified using a Phenomenex Kinetex C_18_ column measuring 100 × 2.1 mm with a particle size of 1.7 μm. The results were detected using a TSQ Quantum Ultra triple quadrupole mass spectrometer. The efficiency of extraction and MS ionization is normalized by spiked deuterated internal references D3-NMT and D6-DMT [25].

## 6. Monitoring the INMT Activity Using Real-Time Assays

**Figure 7:**
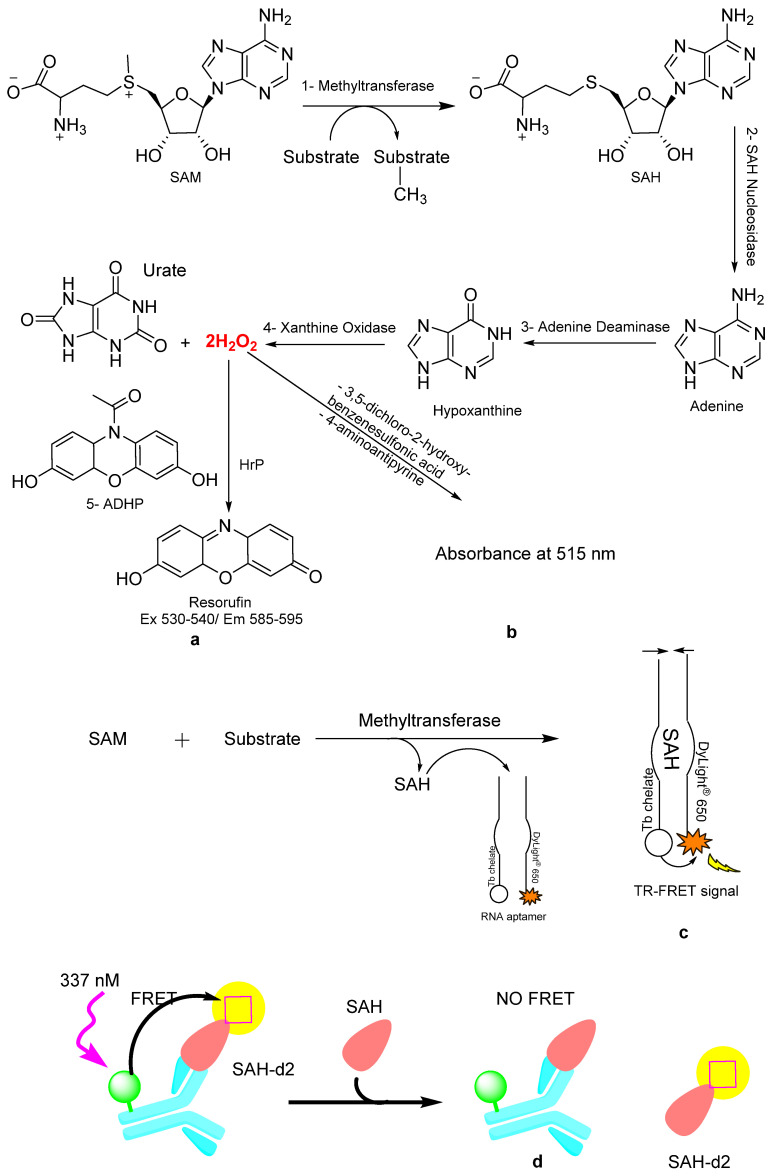
Scheme representation of the methyltransferase real-time assays. (**a**) Fluorometric assay, the ADHP in the presence of horseradish peroxidase (HrP) and H_2_O_2_, is converted to a highly fluorescent compound, resorufin. (**b**) Colorimetric assay: the rate of H_2_O_2_ production is quantified by measuring absorbance at 515 nm with the reagents, 3,5-dichloro-2-hydroxybenzenesulfonic acid, and 4-aminoantipyrine. (**c**) TR-FRET assay: the produced SAH allows the combination of the split aptamer and generates a TR-FRET signal. (**d**) HTRF EPIgeneous^TM^ methyltransferase assay detection step. The detection is based on a competitive immunoassay between the produced SAH and d2-coupled SAH antibody labeled with Lumi4-Tb.

Several methyltransferase assays designed for high-throughput screening applications are commercially available. For selecting the most convenient assay for specific research needs and the available facilities, this section highlights the key features and main differences among the available real-time assays (Figure 7).

### 6.1. Methyltransferase Colorimetric Assay

This assay is compatible with all purified methyltransferases that utilize SAM as a methyl donor [28]. It is an enzyme-coupled continuous assay used to measure the activity of SAM-dependent methyltransferases. The transfer of the methyl group from SAM to the substrate generates the SAH, which is a methyltransferase (MT) inhibitor. To prevent the accumulation of SAH and inhibition of the MT, the produced SAH is rapidly hydrolyzed by SAH nucleosidase into S-ribosyl homocysteine and adenine. This hydrolysis of SAH will allow the MT to continue methylation of the substrate without feedback inhibition. The resulting adenine is quantitatively converted to hypoxanthine by its deaminase. The xanthine oxidase in the enzyme mixture will oxidize the hypoxanthine into urate and liberate H_2_O_2_. The absorbance at 515 nm every 30–60 s for 15–30 min is used to measure the rate of H_2_O_2_ production in the presence of the supplied colorimetric reagents, 3,5-dichloro-2-hydroxybenzenesulfonic acid and 4-aminoantipyrine (Figure 7b).

### 6.2. Methyltransferase Fluorometric Assay

In this assay (Figure 7a), the produced H_2_O_2_ reacts with 10-acetyl-3,7-dihydroxyphenoxazine (ADHP) to form a highly fluorescent compound, resorufin. The fluorescence is recorded by measuring at an excitation wavelength of 530–540 nm and an emission wavelength of 585–595 nm [29].

### 6.3. Apta Fluor SAH Methyltransferase TR-FRET Assay

A universal assay has been developed to monitor all enzymes that convert SAM to SAH, specifically designed for high-throughput screening (see Figure 7c). The reagent consists of two halves of a short sequence of SAH-binding RNA. One of these halves is labeled with DyLight^®^650, while the other half is labeled with terbium (Tb) chelate [26]. The produced SAH facilitates the reassembly of the two aptamers to generate a TR-FRET signal. The generated TR-FRET is measured, and the 665/620 nm ratio is plotted versus the enzyme concentration. The subsequent step is preparing a standard curve for SAM/SAH, which can be used for quantitative conversion of the 665/620 nm ratio to SAH units.

### 6.4. HTRF EPIgeneous^TM^ Methyltransferase Assay

The assay protocol is divided into three main steps on a white plate. First, an enzyme reaction is utilized to generate SAH. Second, the detection phase (Figure 7d) involves competition between SAH and a labeled SAH-d2 for binding to the anti-SAH antibody tagged with Tb-cryptate, resulting in a decreased HTRF signal when SAH displaces SAH-d2. This signal is inversely proportional to the concentration of SAH produced, with fluorescence recorded at 665 nm and 620 nm, after the excitation wavelength of 337 nm. The HTRF is calculated from the 665/620 nm ratio, allowing the quantitative conversion to the corresponding SAH concentration [27].

### 6.5. MTase-Glo™ Assay

MTase-Glo™ bioluminescence-based assay is a universal technique used for measuring and detecting the change in the activity of protein (all classes), DNA, and RNA methyltransferases in high-throughput screening applications [96,97]. The methyltransferases transfer the methyl group from the methyl donor (SAM) to the substrate, producing SAH. After the reaction, the MTase-Glo™ reagent is added to convert SAH into ADP. The ADP is then converted into ATP after adding the MTase-Glo™ detection solution. The resulting ATP is detected through a luciferase reaction. The luminescence is measured with a compatible plate reader. The measured luminescence is correlated to the SAH using a prepared standard curve of SAH.

## 7. Conclusions

This review highlights recent research findings on INMT, focusing on its characterization, associated diseases, inhibitors, and the in vitro assay designed to measure SAM-based methyltransferase activity. INMT produces various endogenous compounds, such as DMT, NMT, and bufotenine, by catalyzing the methylation of indoleamines using SAM as a methyl donor. Additionally, methylation of certain chemical compounds, such as selenium and tellurium, serves as a detoxification pathway. The structure of human INMT (hINMT) has been elucidated, revealing the binding site and interactions with the ligand “SAH”. The human INMT gene is located on chromosome 7 and encodes a protein that exhibits variations, such as the substitution of histidine at position 46 with proline (His46Pro), an example of a single-nucleotide polymorphism (SNP), which may lead to Hirschsprung’s disease. Polymorphisms could also affect DMT levels through altered methylation, which could be linked to schizophrenia. On the other hand, altered INMT expression might play a role in carcinogenesis; reduced INMT levels have been observed in lung adenocarcinoma, and INMT downregulation has been observed in prostate and liver cancers. Our current understanding of INMT’s structural properties and mechanisms remains limited, with only one crystal structure with PDB ID 2A14 available. Furthermore, the biochemical regulation and small molecule control of INMT activity are not yet understood. Detection of the endogenous level of DMT poses a great challenge because it is affected by various physiological factors, along with the report that INMT is not essential for tryptamine methylation in rats. The precise physiological role of INMT has yet to be confirmed, and no definitive relationships have been established due to incomplete characterization. The widespread expression of INMT across organs indicates its pleiotropic importance, but its specific functions remain less thoroughly investigated. Therefore, more studies are still required to understand the INMT substrate recognition mechanisms. Comprehensive investigations should be considered to correlate INMT expression with different disorders and its contribution to metabolism. Ultimately, investigating other biological activities of INMT inhibitors could provide helpful insights into INMT’s true function and will enhance drug development and lead to more effective therapies.

## Figures and Tables

**Table 1:** INMT inhibition assays.

Method	Assay	Description	Reference
M1	Radiometric (in vitro)	^14^C-SAM is used as the methyl donor and tryptamine as the substrate. Formation of ^14^C-methylated tryptamine (^14^C-NMT) is quantified by liquid scintillation counting.	[1]
M2	Fluorometric	N-methylation of N-methylserotonin (NMS) to bufotenine. The resulting bufotenine was derivatized with O-phthalaldehyde (OPT). The fluorescent bufotenine-OPT adduct was then quantified using fluorescence detection.	[20]
M3	Radiometric (in vivo)	Intravenous administration of radiolabeled ^14^C-NMT. After incubation, tissues and plasma were collected, and the radiolabeled product ^14^C-DMT was isolated, quantified by liquid scintillation (LS) counting, and confirmed using reverse isotope dilution analysis.	[93]

**Table 2:** Summary of natural (endogenous) and synthetic inhibitors of indolethylamine-N-methyltransferase (INMT).

Compound ID	Scaffold/Class	Inhibitory Potency	Inhibition Type	Selectivity	Reference
DMT (**1**)	Tryptamine derivative	IC_50_ = 67 μM	Competitive/Non-competitive	Selective	[1,7]
SAH (**2**)	SAM analog	K_i_ = 10 µM	Competitive	Selective	[20]
PDAT (**3**)	Tryptamine derivative	K_i_ = 84 μM	Non-competitive	Selective	[7]
Salsolinol (**4**)	Isoquinoline	97% inhibition at 0.1 mM	Competitive	N/A	[1]
Chloroquine (**5**)	Quinoline	98% inhibition at 0.1 mM	Competitive	N/A	[1]
Harmalol (**6**)	β-Carboline	89% inhibition at 1 mM	Competitive	N/A	[1]
Naratriptan (**7**)	Triptan	IC_50_ = 147 μM	Competitive	Non-selective	[1]
Sumatriptan (**8**)	Triptan	IC_50_ = 370 μM	Competitive	Non-selective	[1]
Zolmitriptan (**9**)	Triptan	IC_50_ = 483 μM	Competitive	Non-selective	[1]
1,7-diaminoheptane (**13**)	Aliphatic diamine	84.9% inhibition at 10 mM	Competitive	Non-selective	[23]
1,8-diaminooctane (**14**)	Aliphatic diamine	94.3% inhibition at 10 mM	Competitive	Non-selective	[23]
DBN (**18**)	Bicyclic amidine	IC_50_ = 20 μM	Non-competitive	Selective	[93]
3,4,6,7,8,9-hexahydro-2H-pyrido [1,2-a]pyrimidine (**19**)	Bicyclic amidine	IC_50_ = 0.7 μM	Non-competitive	Selective	[22]
2,3,5,6-tetrahydro-7H-pyrrolo [1,2-a]imidazole (**20**)	Bicyclic amidine	IC_50_ = 0.04 μM	Non-competitive	Selective	[22]
2,3,5,6,7,8-hexahydroimidazo [1,2-a]pyridine (**21**)	Bicyclic amidine	IC_50_ = 0.17 μM	Non-competitive	Selective	[22]
2,3,5,6-Tetrahydro-8H-imidazo [2,1-c][1,4]thiazine (**22**)	Bicyclic amidine	IC_50_ = 0.5 μM	Non-competitive	Selective	[22]
3-methylthiazolidin-2-imine (**23**)	Monocyclic amidine	IC_50_ = 0.8 μM	Non-competitive	Selective	[22]
3-ethylthiazolidin-2-imine (**24**)	Monocyclic amidine	IC_50_ = 0.2 μM	Non-competitive	Selective	[22]
2-imino-N-methylthiazolidin-3-amine (**25**)	Monocyclic amidine	IC_50_ = 1.5 μM	Non-competitive	Selective	[22]
3-methylthiazol-2(3H)-imine (**26**)	Monocyclic amidine	IC_50_ = 2 μM	Non-competitive	Selective	[22]
1-methylpyrrolidin-2-imine (**27**)	Monocyclic amidine	IC_50_ = 1.5 μM	Non-competitive	Selective	[22]
1-ethylpyrrolidin-2-imine (**28**)	Monocyclic amidine	IC_50_ = 1.6 μM	Non-competitive	Selective	[22]
3-methyl-1,3-thiazinan-2-imine (**29**)	Monocyclic amidine	IC_50_ = 2 μM	Non-competitive	Selective	[22]
3-ethyl-1,3-thiazinan-2-imine (**30**)	Monocyclic amidine	IC_50_ = 1.8 μM	Non-competitive	Selective	[22]
3-methyl-3,6-dihydro-2H-1,3-thiazin-2-imine (**31**)	Monocyclic amidine	IC_50_ = 2 μM	Non-competitive	Selective	[22]
1-methyl-5,6-dihydropyridin-2(1H)-imine (**32**)	Monocyclic amidine	IC_50_ = 0.9 μM	Non-competitive	Selective	[22]
N^6^-methyl-3-deazaadenosyl-L-homocysteine (**34**)	Homocysteine derivatives	N/A	Competitive	Selective	[94]
N^6^-methyladenosyl-L-homocysteine (**35**)	Homocysteine derivatives	IC_50_ = 17.4 μM	Competitive	Selective	[94]
Imipramine (**36**)	Tricyclic antidepressant	IC_50_ = 166 μM	Competitive	N/A	[1]

N/A, not applicable; inhibition potency, represents IC_50_ or K_i_ or percentage inhibition.

## Abbreviations

The following abbreviations are used in this manuscript:

INMT	Indolethylamine N-methyltransferase
DMT	N,N-dimethyltryptamine
NMT	N-methyltransferase
SAM	S-adenosyl-L-methionine
MAO	Monoamine oxidase
SAH	S-adenosyl-L-homocysteine
NNMT	Nicotinamide N-methyltransferase
PNMT	Phenylethanolamine N-methyltransferase
CRPC	Castration-resistant prostate cancer
NSCLC	Non-small cell lung cancer
UCEC	Uterine corpus endometrial carcinoma
HSCR	Hirschsprung’s disease
ALS	Amyotrophic lateral sclerosis
